# Determination of the median effective dose (ED_50_) of bupivacaine and ropivacaine unilateral spinal anesthesia: Prospective, double blinded, randomized dose-response trial

**DOI:** 10.1007/s00101-017-0370-9

**Published:** 2017-10-25

**Authors:** WeiBing Wang, YuanHai Li, AiJiao Sun, HongPing Yu, JingChun Dong, Huang Xu

**Affiliations:** 1 Department of Anesthesiology, The First Affiliated Hospital of Anhui Medical University, Jixi Road 218th, 230031 HeFei, China; 2 Department of Anesthesiology, The Affiliated AnQing Municipal Hospital of Anhui Medical University, AnQing, China; 3 Department of Cardiovasology, The Affiliated AnQing Municipal Hospital of Anhui Medical University, AnQing, China

**Keywords:** Hip replacement arthroplasty, Geriatrics, Prospective study, Cerebrospinal fluid, Outcome, Hüftprothesenoperation, Geriatrie, Prospektive Studie, Cerebrospinalflüssigkeit, Outcome

## Abstract

**Background:**

Unilateral spinal anesthesia (USpA) has been reported to potentiate spinal anaesthesia and is used in geriatric patients. The purpose of this study was to determine the median effective dose (ED_50_) of 0.5% hypobaric bupivacaine and 0.5% hypobaric ropivacaine USpA for geriatric patients (age ≥ 70 years) undergoing elective hip replacement surgery.

**Methods:**

A total of 60 geriatric patients (age ≥ 70 years) undergoing elective hip replacement surgery were enrolled in this study. The patients were randomized into 2 groups to receive either intrathecal 0.5% hypobaric bupivacaine USpA (group B) or 0.5% hypobaric ropivacaine USpA (group R). Effective anesthesia was defined as a T10 sensory blockade level maintained for more than 60 min, and a Bromage score of 3 on the operation side within 10 min after injection with no additional epidural anesthetic required during surgery. The ED_50_ of 0.5% hypobaric bupivacaine and 0.5% hypobaric ropivacaine was calculated using the Dixon and Massey formula.

**Results:**

No significant differences were found between the two groups in terms of demographic data. The ED_50_ of 0.5% hypobaric bupivacaine USpA was 4.66 mg (95% confidence interval CI 4.69–4.63 mg) mg and that of 0.5% hypobaric ropivacaine USpA was 6.43 mg (95% CI 6.47–6.39 mg) for geriatric patients undergoing hip replacement surgery.

**Conclusion:**

We find the ED_50_ were lower, and the ED50 of 0.5% hypobaric bupivacaine and ropivacaine was 4.66 mg (95% CI 4.69–4.63 mg) and 6.43 mg (95% CI 6.47–6.39 mg), respectively, for USpA in geriatric patients (age ≥ 70 years) undergoing elective hip replacement surgery.

## Zusammenfassung

**Hintergrund:**

Die unilaterale Spinalanästhesie (USpA) soll die Spinalanästhesie potenzieren und wird bei geriatrischen Patienten verwendet. Zweck dieser Studie war die Bestimmung der mittleren effektiven Dosis (ED_50_) einer USpA mit 0,5%igem hypobarem Bupivacain und 0,5%igem hypobarem Ropivacain bei geriatrischen Patienten (Alter ≥ 70 Jahre), die sich einer elektiven Hüftprothesenoperation unterzogen.

**Methoden:**

Sechzig geriatrische Patienten (Alter ≥ 70 Jahre), die sich einer elektiven Hüftprothesenoperation unterzogen, wurden in diese Studie eingeschlossen. Die Patienten wurden in 2 Gruppen randomisiert, um entweder eine intrathekale USpA mit 0,5%igem hypobarem Bupivacain (Gruppe B) oder 0,5%igem hypobarem Ropivacain zu erhalten. Eine effektive Anästhesie wurde definiert als ein sensorisches Blockadeniveau von T10, das mehr als 60 min aufrechterhalten werden konnte, sowie ein Bromage-Score von 3 innerhalb von 10 min nach der Injektion, ohne dass eine zusätzliche epidurale Anästhesie während der Operation benötigt wurde. Die ED_50_ von 0,5%igem hypobarem Bupivacain und 0,5%igem hypobarem Ropivacain wurde mit der Formel nach Dixon und Massey berechnet.

**Ergebnisse:**

Bezüglich der demographischen Daten wurden keine signifikanten Unterschiede zwischen den beiden Gruppen gefunden. Die ED_50_ einer USpA mit 0,5%igem hypobarem Bupivacain betrug 4,66 (95% Konfidenzintervall [CI] 4,69–4,63) mg, und die ED_50_ einer USpA mit 0,5%igem hypobarem Ropivacain lag bei 6,43 (95% CI 6,47–6,39) bei geriatrischen Patienten (Alter ≥ 70 Jahre), die sich einer Hüftprothesenoperation unterzogen.

**Schlussfolgerung:**

Die ED_50_ einer USpA mit 0,5%igem hypobarem Bupivacain und Ropivacain betrug 4,66 (95% CI 4,69–4,63) mg bzw. 6,43 (95% CI 6,47–6,39) bei geriatrischen Patienten (Alter ≥ 70 Jahre), die sich einer elektiven Hüftprothesenoperation unterzogen.

## Background

Unilateral spinal anesthesia (USpA) is a cost-effective and rapidly performed anesthetic technique. An exclusively unilateral block only affects the sensory, motor and sympathetic functions on one side of the body and provides the advantages of a spinal block without the typical adverse side effects of a bilateral block. In particular, the lack of hypotension makes USpA suitable for geriatric patients [1]. Hip fractures and femoral head necrosis are global public health problems in geriatric patients. There are increasing trends in the incidences of these issues due to both the increasing average life expectancy, and the increasing incidence of osteoporosis. Over 90% of hip fracture patients are older than 65 years and have pre-existing medical comorbidities [2, 3]. Much of the currently available evidence suggests that a comprehensive medical approach with emphasis on regional anesthesia can prove beneficial to patients and the healthcare system [4]. Recently, a systematic review and meta-analysis found that neuraxial anesthesia is associated with a reduced in-hospital mortality and length of hospitalization [5].

Spinal anesthesia is a routinely used anesthetic technique in geriatric patients undergoing hip replacement surgery in the lateral decubitus position. As such, hypobaric USpA can be used for these surgeries without changing the patient^’^s position. Because both the functional reserve and ability to compensate for physiological stresses are reduced in elderly patients [6], excessive local anesthetics can still result in hypotension and bradycardia. Thus, optimizing the dose of hypobaric USpA is important in geriatric patients. A recent study suggested that the 365-day chance of mortality was marginally lower in patients with spinal/neuraxial anesthesia than with general anesthesia [7]. These authors recommended preventing hypotension associated with spinal blocks, hypoxia and anemia, which may lead to the occurrence of perioperative adverse events [8]. The use of USpA is safe, the dose of local anesthetic is lower, and a major advantage of USpA is hemodynamic stability [9].

A recent meta-analysis did not find any significant differences in the 30-day mortality or postoperative complications of patients who received general anesthesia versus spinal anesthesia for the surgical repair of a hip fracture [10]; however, spinal anesthesia was related to significantly decreased early mortality, and reduced the cases of deep vein thrombosis, acute postoperative confusion, myocardial infarction, pneumonia, and fatal pulmonary embolisms and in addition, postoperative hypoxia, and duration of hospitalization were reduced [11, 12]. Low dose local anesthetic solutions administered via a pencil-point needle and slow intrathecal injection have been reported to result in satisfactory USpA, which should also minimize the cardiovascular effects of spinal blocks [13, 14].

## Methods

### Design

We conducted a prospective, double-blinded, up-down sequential allocation study to determine the median effective dose (ED_50_) of intrathecally administered 0.5% hypobaric bupivacaine and 0.5% hypobaric ropivacaine for USpA in geriatric patients (age ≥ 70 years) undergoing elective hip replacement surgery.

### Subjects and setting

A total of 60 geriatric patients (age ≥ 70 years) undergoing elective hip replacement surgery were enrolled in the current study. The study was approved by the medical ethical committees of The Affiliated AnQing Municipal Hospital of Anhui Medical University (approval date: 26 December 2015). All patients provided written informed consent. This study was registered in the Chinese Clinical Trial Registry (ID: ChiCTR-OOR-16008755). The registration information can be found on the following website: http://www.chictr.org.cn/searchprojen.aspx.

The exclusion criteria were contraindications to spinal anesthesia, including local infections at the puncture site, bacteremia, severe hypovolemia, coagulopathy, severe stenotic valvular disease, infections at the site of the procedure, and intracranial hypertension. Relative contraindications included progressive degenerative (demyelinating) neurological diseases (e.g. multiple sclerosis), low back pain, and sepsis. Comorbidities predisposing patients to severe hypotension and/or a severely altered mental status were exclused also.

### Study protocol

Patients were randomized into one of two groups, the 0.5% hypobaric bupivacaine group (group B, *n* = 30) and the 0.5% hypobaric ropivacaine group (group R, *n* = 30), based on a computer-generated random number list (Microsoft, Excel), which was kept in a sealed opaque envelope before the start of the study (prepared by AJS). All patients underwent preoperative fasting for 8 h and water deprivation for more than 4 h. On arrival to the operating theatre, standard monitoring was applied via automated non-invasive blood pressure measurements, electrocardiography and pulse oximetry. The baseline mean arterial blood pressure (MAP) and heart rate (HR) were monitored throughout the operation. The patient was administered 8 ml/kg body weight of lactated Ringer’s solution for 10 min via a 16-gauge cannula placed in a forearm vein. The infusion speed was then adjusted to 8 ml·kg^−1^·h^−1^.

All patients were placed in the lateral decubitus position with the operation side in the upper position. A combined spinal-epidural procedure was performed (the spinal procedure was performed by WBW, an associate chief physician of anesthesiology). Dural puncture was performed using a 25-gauge Quincke point needle (Spinocan, Braun Melsungen, Germany) inserted in the midline at the L 2/3 interspace under aseptic conditions. After dural puncture, the spinal needle was injected into the subarachnoid space. After cerebrospinal fluid (CSF) appeared in the spinal needle hub, the needle hole was turned upwards and a dose of 0.5% hypobaric local anesthetic was injected at a rate of 0.1 ml/s without barbotage via the up and down method. The spinal anesthesia needle was then withdrawn, and 3 cm of the epidural catheter was inserted into the epidural space. The lateral decubitus position was maintained until the end of surgery. The mixed solutions for spinal anesthesia were prepared before anesthesia by an anesthesia assistant (HPY), who did not participate in the subsequent patient assessment. The solutions were administered by a second attending anesthesiologist (HX or JCD), who remained blinded to the mixed solution contents. The mixed solution for patients in group B was as follows: 2.0 ml of 0.75% bupivacaine (ZHAOHUI Company, Shanghai, China; production batch: 73150405) diluted with sterile distilled water to a total volume of 3 ml. The mixed solution for patients in group R was as follows: 2.0 ml of 0.75% ropivacaine (AstraZeneca AB, Sweden; production batch: LASC) diluted with sterile distilled water to a total volume of 3 ml. The density of the hypobaric ropivacaine and hypobaric bupivacaine solutions was determined to be 0.9980, and 0.9976, respectively.

Previous research investigating the ED_50_ of intrathecal ropivacaine and bupivacaine for lower limb surgery in Chinese patients found that the ED_50_ was 8.41 mg (95% confidence interval CI: 7.15–9.67 mg) for ropivacaine and 5.5 mg (95% CI: 4.90–6.10 mg) for bupivacaine. The relative anesthetic potency ratio was 0.65 (95% CI: 0.54–0.80) for ropivacaine/bupivacaine [15]. Therefore, in our study, we used a lower dose of hypobaric local anesthetic for USpA. The first patient in group B received 0.5% hypobaric bupivacaine 6.0 mg, and the first patient in group R received 0.5% hypobaric ropivacaine 8.0 mg. The testing interval in each group was 0.5 mg. If the response of the previous patient was effective, the dose of intrathecal hypobaric local anesthetic for the next patient was decreased by 0.5 mg in that group. Conversely, if the response of the patient was ineffective, the dose of intrathecal hypobaric local anesthetic for the next patient was increased by 0.5 mg in that group. An effective outcome was defined as a T10 sensory blockade level maintained for more than 60 min, and a Bromage score of 3 on the operation side within 10 min after injection otherwise the outcome was ineffective.

### Measurements

Throughout the monitoring period, if the MAP was determined to have dropped by more than 20% of the preoperative basal values, a rapid intravenous (i. v.) infusion of lactated Ringer’s solution was administered, and if necessary, a 10 mg i. v. bolus of ephedrine was administered at 1 min intervals. If the HR fell below 50 beats/min, a 0.5 mg i. v. bolus of atropine was administered. If nausea and vomiting were observed, 10 mg of i. v. metoclopramide was administered. An SpO_2_ (peripheral capillary oxygen saturation) below 92% was evaluated as hypoxia, and 4 l·min^−1^ oxygen was administered via a face mask. The sensory blockade level was determined by assessing the loss of pain sensation along the operation side using a 20-gauge sterilized needle. If the T10 sensory blockade level was not achieved within 10 min an epidural supplement of 2% lidocaine was administered to maintain a T10 sensory level.

The side effects and complications of spinal anesthesia include the following: shivering, nausea and vomiting, post-dural puncture headache (PDPH) and respiratory depression (defined as a breath rate < 12 bpm or an SpO_2_ < 90%) during surgery and the first 24 h after surgery. All side effects and complications were recorded by an anesthesia assistant. The motor block was evaluated with a modified Bromage scale as follows: 0 no motor block, 1 hip flexion with extended leg blocked, 2 knee flexion blocked, and 3 complete motor block. The onset time of motor block was defined as the time between spinal injection and a Bromage score of 1 being reached. The time of Bromage score regression was defined as the period between the time of motor block from 3 to 1.

### Statistical analysis

The sample size estimation was calculated using G*Power software (Heinrich-Heine-University, Düsseldorf, Germany). An estimated “average” SD of difference of the ED_50_ of intrathecal hypobaric bupivacaine and ropivacaine between groups is 0.5 mg, and power was given at 0.95 to detect a difference of 1.6 SD (0.8 mg) at *P* < 0.05. A minimum of 12 subjects were then necessary in each group. Because the Dixon and Massey technique requires the sample size to be approximately twice this number (as the estimations of ED_50_, SE and 95% CI are based on the number and distribution of the lesser occurring outcome, which will be approximately 50% of the observations); therefore, 30 patients per group was a sufficient sample size.

Statistical analyses were carried out using SPSS 17.0 for Windows (SPSS, Chicago, IL). Data are expressed as the mean (standard deviation SD), median (range), or count/number. The means were compared using a one-way ANOVA, while the medians (ranges) were analyzed by a one-way Kruskal-Wallis analysis. The ED_50_ values for the hypobaric local anesthetics were determined according to the up and down sequential method of Dixon and Massey [16], A *P*-value < 0.05 was considered statistically significant.

## Results

**Fig. 1:**
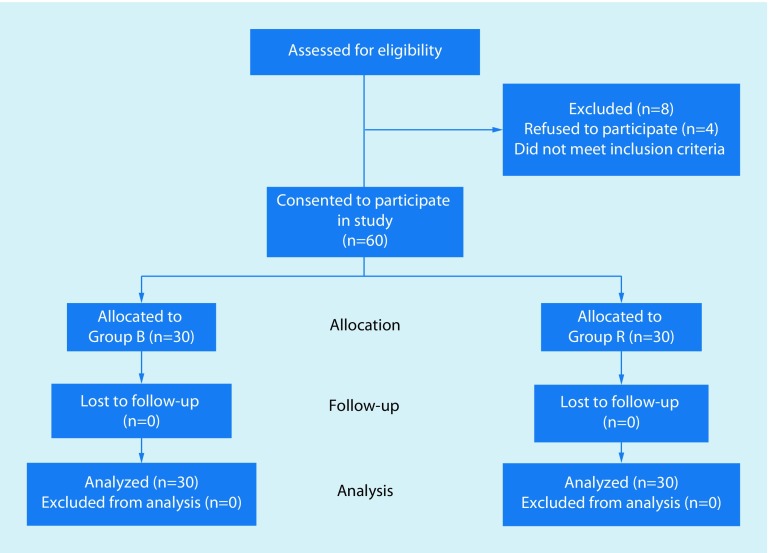
CONSORT diagram of the patient selection procedure

The CONSORT diagram of the present study is showed in Fig. 1. A total of 68 patients were assessed for eligibility, among them 60 patients were enrolled and randomly assigned into group B (*n* = 30) or group R (*n* = 30). All 60 patients finished the study and were included in the final analysis.

The patient demographic characteristics (age, weight, gender and height), American Society of Anesthesiologists (ASA) classification, surgery duration, and sensory block duration are shown in Table 1. There were no significant differences between the groups.

**Table 1:** Demographic characteristics of all patients, ASA classification, surgery duration, and sensory block duration

Variable	Group B (*n* = 30)	Group R (*n* = 30)	*P*-value
Age (years)	74.9 ± 8.6	72.4 ± 7.3	0.889^a^
Height (cm)	168.3 ± 6.5	171.9 ± 4.6	0.965^a^
Weight (kg)	71.8 ± 12.1	69.5 ± 9.2	0.812^a^
Gender (M/F)	13/17	20/10	0.664^b^
ASA (I/II/III)	3/24/3	2/25/3	0.589^b^
Surgery duration (min)	51.4 ± 12.8	56.3 ± 9.7	0.632^a^
Sensory block duration (min)	102 ± 18	96 ± 22	0.580^a^

Data are expressed as the mean ± SD

*ASA* American Society of Anesthesiologists*, group B* hypobaric bupivacaine group, *group R* hypobaric ropivacaine group, *M* male, *F* female

^a^ *P*-value: Student’s *t* test

^b^ *P*-value: χ^2^-test

When the mean HR and MAP measurements obtained throughout the monitoring period were compared with the baseline values, no significant differences were found (*P* = 0.832 and 0.417, respectively) and no significant difference was found between the baseline SpO_2_ measurements and those obtained throughout the monitoring period (*P* = 0.265). The onset time and regression time of motor block, and sensory blockade level are shown in Table 2.

**Table 2:** Onset time and regression time of motor block, and sensory blockade level

Time (min)	Group B	Group R	*P*-value
Onset time	6.2 ± 3.5	7.4 ± 3.5	0.650
Regression time	92 ± 22	86 ± 26	0.760
Sensory blockade level	T11 ± 1.2	T10 ± 1.6	0.920

*P*-value: Student’s *t* test

The incidences of USpA side effects, such as hypotension, nausea and vomiting, shivering, PDPH, urinary retention, respiratory depression and number of ephedrine bolus administrations during the perioperative period, are shown in Table 3.

**Table 3:** Side effects of USpA and number of ephedrine bolus administrations

	Group B (*n* = 30)	Group R (*n* = 30)	*P*-value
Hypotension	4 (13.3)	6 (20.0)	0.63
Nausea and vomiting	3 (10.0)	4 (13.3)	0.75
Shivering	5 (16.7)	6 (20.0)	1.00
Number of ephedrine bolus administrations	4 (13.3)	6 (20.0)	0.63
PDPH	0	0	–
Respiratory depression	0	0	–
Urinary retention	0	0	–

Data are presented as the number (%) or the mean ± SD

*PPDH* post-dural puncture headache

Hypotension: the MAP dropped by more than 20% of the preoperative basal values

Respiratory depression: an individual’s respiration has a rate below 12 breaths per min and/or an SpO_2_ below 92%

*P*-value: χ^2^-test

**Fig. 2:**
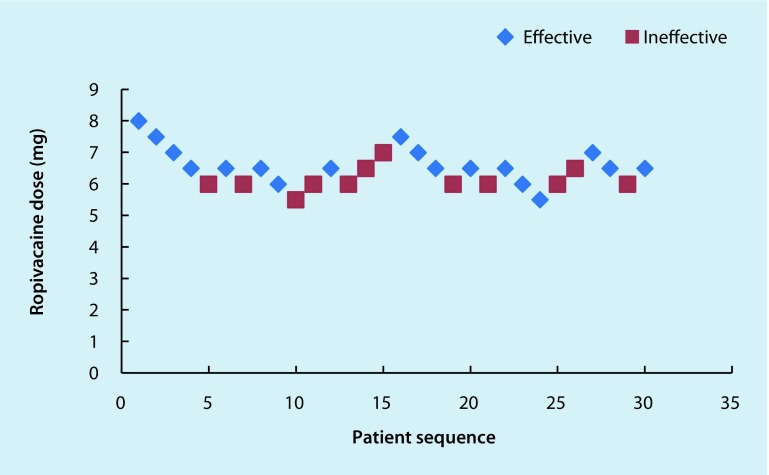
The ED_50_ of intrathecal 0.5% hypobaric ropivacaine USpA in geriatric patients undergoing hip replacement surgery, as determined using the Dixon and the Massey up-down sequential method was 6.43 mg (95% CI: 6.47–6.39 mg). In this group 12 patients needed an epidural supplement of 2% lidocaine to maintain a T10 sensory level, and 4 patients exhibited nausea and vomiting after the administration of 2% lidocaine into the epidural space. Individual responses to intrathecal hypobaric ropivacaine at specific doses. The *red squares* represent an ineffective response to the corresponding dose of intrathecal hypobaric ropivacaine for spinal anesthesia. The *blue diamonds* represent an effective response to the corresponding dose of intrathecal hypobaric ropivacaine for spinal anesthesia

**Fig. 3:**
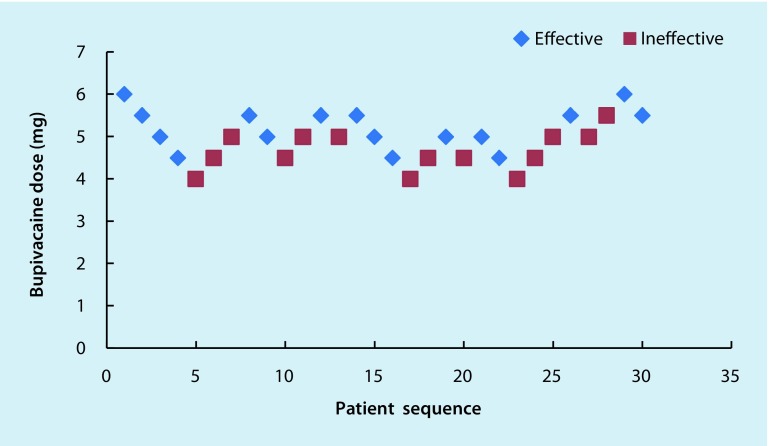
The ED_50_ of intrathecal 0.5% hypobaric bupivacaine USpA in geriatric patients undergoing hip replacement surgery, as determined using the Dixon and the Massey up-down sequential method was 4.66 mg (95% CI: 4.69–4.63 mg). In this group 14 patients needed an epidural supplement of 2% lidocaine to maintain a T10 sensory level, and 3 patients exhibited nausea and vomiting after the administration of 2% lidocaine into the epidural space. Individual responses to intrathecal hypobaric bupivacaine at specific doses. The *red squares* represent an ineffective response to the corresponding dose of intrathecal hypobaric bupivacaine for spinal anesthesia. The *blue diamonds* represent an effective response to the corresponding dose of intrathecal hypobaric bupivacaine for spinal anesthesia

The sequences of effective and ineffective outcomes in the two groups are shown in Figs. 2 and 3.

The ED_50_ of 0.5% hypobaric bupivacaine USpA was 4.66 mg (95% CI: 4.69–4.63 mg) and that of 0.5% hypobaric ropivacaine USpA was 6.43 mg (95% CI: 6.47–6.39 mg) in geriatric patients (age ≥ 70 years) undergoing hip replacement surgery.

## Discussion

While the relative contribution of anesthesia to the outcome of hip replacement surgery remains uncertain, better outcomes are associated with standardized practices. A key objective of the current study was to present our practices with the hope of developing a consensus regarding better practices for reducing side effects resulting from subarachnoid local anesthetic administration for hip surgery in the geriatric population. These results will help individual anesthetists and departments of anesthesia to improve the management of those geriatric populations.

A rare case was reported in one study in which there was a 45-min delay between the administration and onset of action of a subarachnoid blockade in a 103-year-old female patient. This patient received an injection of hyperbaric bupivacaine (1.5 ml of 0.75%, 11.25 mg), with 15 µg of fentanyl into the subarachnoid space [17]. The authors believed that the baricity of anesthetic solutions might be one factor affecting the achievement of successful spinal anesthesia.

The results of the present study show that the addition of sterile distilled water to anesthetic solutions changed the density of the anesthetics, resulting in different required hypobaric anesthetic doses for lower extremity surgery. In this study, the density of hypobaric ropivacaine and bupivacaine was 0.9960 and 0.9976, respectively. As the average density of CSF is 1.0003 ± 0.0003, we believe that a low density is conducive to the sufficient distribution of anesthetics in CSF. In the study, temperature-dependent densities of the isobaric local anesthetics bupivacaine and ropivacaine were found to be hypobaric at body temperature [18]; therefore, the distribution of anesthetics in CSF would be more suitable for hypobaric anesthetics than for isobaric and hyperbaric anesthetics. The use of USpA yields more stable cardiovascular parameters than conventional bilateral spinal blockades [19] and the aim is to limit the distribution of the spinal block to the side of the operation. In our study, USpA was achieved using small doses of local anesthetic solutions injected by a directional, pencil-point needle with the patient in the lateral decubitus position. In addition, a hypobaric solution was used, so the patient’s position did not need to be changed, and the lateral position was maintained until the end of surgery, which led to better patient and surgeon acceptance. The sensory block level, following the injection of local anesthetics is influenced by various factors, including age, height, local anesthetic concentration and specific gravity, patient posture, needle bevel direction, drug dosage, barbotage, and injection site [20].

Compared with previous research [15, 21] our study used a lower dose. The mechanism underlying this difference might be related to the injection of the local anesthetic towards the upper side of the patient with the Quincke spinal needle. This approach probably increased the mixing of the local anesthetic molecules with the CSF, and the local anesthetic probably easily diffused through the CSF because of its lower viscosity. In addition, the hypobaric local anesthetics were used to cause a unilateral spinal block, which requires less local anesthetic.

Selective USpA with 0.5% hyperbaric bupivacaine injected at 0.33 ml/min up to a maximum dose of 5 mg was found to be a useful approach for ambulatory lower limb surgery. This approach resulted in a more stable hemodynamic course and fewer adverse events than those resulting from bilateral blocks. The median decrease in systolic blood pressure was 6 mm Hg [22], and several authors have reported low incidence rates of intraoperative hypotension with low dosages of subarachnoid bupivacaine and levobupivacaine for hip fracture surgery in elderly patients [23]. Furthermore, we found no significant differences between the MAP and HR values measured during surgery and the corresponding baseline values in our study. The mechanism underlying these findings might be related to the lower local anesthetic dose not leading to excessive cephalic diffusion and in addition the hypobaric USpA limits the block region, such that the vascular resistance was blocked only on one side.

Hyperbaric USpA is a known technique to obtain stable hemodynamics. Because a hyperbaric unilateral technique can be very painful in cases of traumatic hip fracture, a low dose, low volume, hypobaric USpA may be an adequate alternative. Hypobaric USpA is a simple technique, produces satisfactory operative conditions and induces very little hemodynamic change in the elderly population [24], thus, the side effects maybe decrease.

Redistribution of intrathecal local anesthetics is determined principally by baricity and position of the patient. Hypobaric solutions of local anesthetics are characterized by an unpredictable spread of sensory block. Some studies shown the density of local anesthetics decreases with increasing temperature [18, 25]. In our study, we used low dose hypobaric local anesthetics for USpA, and we did not find unpredictable spread of sensory block, there were no side effects, such as PDPH, respiratory depression and urinary retention.

In all, 12 patients in group R and 14 patients in group B needed an epidural supplement of 2% lidocaine to maintain a T10 sensory level. There was a similar prevalence of nausea and vomiting in each group, and no bradycardia was observed. Of the patients 3 in group B and 4 patients in group R might have experienced nausea and vomiting maybe because of hypotension, due to the administration of 2% lidocaine into the epidural space.

Some studies have shown that using very small doses of hyperbaric bupivacaine together with an intrathecal opioid in hip or knee surgery results in less hypotension than using conventional spinal anesthesia doses, and provides a sufficient block in most elderly patients [26, 27]; however, in those studies, the rates of postoperative nausea and vomiting (PONV) and, urinary retention were higher. Thus we prefer to use low dose hypobaric USpA to prevent PONV and urinary retention that commonly occur with opioids. The average sensory block duration in the 2 groups assessed here was 96—102 min, with no significant difference between the groups.

Our study has some limitations, as follows, which might slightly affect the results:

- Injection controlled by hand instead of by a microperfusion pump might have resulted in a bilateral block in some patients, which would affect the sensory level.
- Some hip fracture patients cannot flex their hip because of pain, which would affect the modified Bromage scale scores.
- Some geriatric patients cannot communicate coherently, which decreases the accuracy of the sensory level assessment.

Although the up and down method has often been used in small samples to determine the ED_50_ of a drug, the ED_95_ cannot be accurately assessed using this approach; therefore, further investigations are required to determine the ED_95_ of bupivacaine and ropivacaine hypobaric USpA in geriatric patients undergoing hip replacement surgery.

## Conclusion

Our study showed that the hypobaric local anesthetics dose required for USpA is low in geriatric patients undergoing hip replacement surgery. The ED_50_ of 0.5% hypobaric bupivacaine USpA was 4.66 mg (95% CI: 4.69–4.63 mg) and that of 0.5% hypobaric ropivacaine USpA was 6.43 mg (95% CI: 6.47–6.39 mg) in geriatric patients (age ≥ 70 years) undergoing hip replacement surgery.
